# Investigation of the disruption of algal biomass with chlorine

**DOI:** 10.1186/s12870-018-1614-9

**Published:** 2019-01-09

**Authors:** Temesgen Garoma, Ramin E. Yazdi

**Affiliations:** Department of Civil, Construction and Environmental Engineering, San Diego State University, 5500 Campanile Drive, San Diego, CA 92182 USA

**Keywords:** Chlorine, Cell disruption, Cell viability, Specific energy, Global warming potential

## Abstract

**Background:**

Algal biofuel has a potential for reducing dependence on fossil fuel while curbing CO_2_ emissions. Despite these potential benefits, a scalable, sustainable, and commercially viable system has not yet been developed. One of the key barriers is the lack of viable methods for disrupting algal biomass for the separation and extraction of bioproducts. This study experimentally investigated the feasibility of using chlorine as an agent for algal biomass disruption.

**Results:**

Chlorine was an effective agent for disrupting algal cell, as demonstrated with cell viability and SEM analyses. For disruption studies conducted using algal suspension at 0.02% solids (0.2 g/L), 90% of the algal cells were disrupted in 6 min at 10 mg/L chlorine dose. Moreover, the results demonstrated that the estimated specific energy requirement, specific cost, and GWP for chlorine were lower than those of the existing methods. The energy requirement for chlorine was 3.73 MJ/ kg of dry algae disrupted, while the requirements for the existing methods ranged from 33 to 860 MJ/ kg of dry algae. The GWP for chlorine was 0.3 kg CO_2_-eq./kg dry algae, while for the existing methods it varied from 5.9 to 369.8 CO_2_-eq./kg dry algae. Despite these advantages, it was observed that residual chlorine reacted with and mineralized the cell contents, which is undesirable.

**Conclusions:**

Future research efforts must be focused on eliminating or reducing the reaction of residual chlorine with cell contents. If this challenge is addressed, chlorine has a potential to be developed into an energy-efficient, cost-effective, and sustainable method for algal biomass disruption. This will in turn will overcome one of the technical bottlenecks, and ultimately increase algal biofuel production and reduce dependence on fossil fuel and curb CO_2_ emissions.

## Background

Algae have emerged as the most promising long-term and sustainable feedstock for biofuel production due to their high productivity rate [1], ability to tolerate a wide range of growth conditions [2], and lack of competition for land and water with food crops [3]. Moreover, CO_2_ sequestration via algae was estimated to be one to two orders of magnitude higher than terrestrial plants [4]. Therefore, algal biofuel has a potential to help reduce dependence on fossil fuel, while curbing CO_2_ emissions.

The current research focus on the algal biofuel arena is on two main algae-to-fuel pathways, namely: (1) algal bioproducts extraction and upgrading [5] and (2) whole algae hydrothermal liquefaction and upgrading [6]. In the former pathway, one of the key barriers is the lack of viable methods for disrupting algal biomass for the separation and extraction of bioproducts [7]. A variety of methods are currently available for algal biomass disruption, including bead milling, high-pressure homogenization, high-speed homogenization, hydrodynamic cavitation, microwave, ultrasonication, pulsed electric field, and Joule heating, among others [7–11]. Most of these methods are adopted from the food industry, where energy-efficiency and cost-effectiveness are less of a factor of viability for a technology since food products can command a high price on the market. Review of scientific literatures on the existing algal biomass disruption methods shows that specific energy requirements vary from 33 megajoule (MJ) per kg of dry algae for hydrodynamic cavitation to 860 MJ per kg of dry algae for pulsed electric field [8]. The energy available by the combustion of the whole algal biomass was estimated to be about 22 MJ per kg of dry [12]. Therefore, the existing biomass disruption methods result in a negative net energy balance. This fact has been already demonstrated through an Energy Return of Investment (EROI) analysis performed for various algal bioproducts extraction and upgrading pathways, resulting in EROIs in the ranges of 9.2 × 10^− 5^ to 0.36 [13].

The energy required for the indentation and disruption of a single algae cell was estimated as 17 picojoule (pJ) with an atomic force microscope [14], which was estimated to be equivalent to 670 J per kg of dry algae cell. This demonstrates that the existing disruption methods are highly inefficient in transferring energy to the algae cells. In hydrodynamic cavitation, the most “efficient” of the existing methods, only about 0.002% of the energy input was used for cell disruption. This clearly shows that any incremental improvement in the efficiencies of the existing biomass disruption methods will not bring about a significant change in the algal biofuel industry.

Chemicals including chlorine, ozone, H_2_O_2_, and surfactants have been proposed as possible alternative cell-disruption agents [15, 16]. However, the studies to date focused on the application of these chemicals for the removal or control of eutrophication, algae from fresh water, algal toxicity, or biofouling [17–19]. The purpose of this study was to evaluate the feasibility of using chlorine for algal biomass disruption. We are not aware of any published or unpublished work in which chlorine was used as a disruption agent for algal biomass. Some of the questions that need to be answered include: What are the impacts of chlorine on algal cell viability, surface morphology, and cell size? Is there enhancement in lipid extraction yield due to algal biomass disruption with chlorine? Will the use of chlorine have any impact on the lipid? This study sought to address these questions.

## Methods

### Experimental approach

The disruption of algal cell with chlorine was investigated using: (1) dilute algal suspension at 0.02% solids and (2) algal paste at 10% solids, both on dry mass basis. The set-up for disruption experiment involving algal suspension consisted of 500 mL Erlenmeyer flasks (Kimble Chase) with a taper PTFE stopper. The working volume of the reactors was set to 400 mL to maintain semi-batch reactors after periodic withdrawal of samples for the various analyses conducted. Amber flasks were used to prevent transmission of light to the algae cultures.

In a typical disruption experiment involving algal suspension, first the algal culture was centrifuged at 10,000 G for 10 min and the supernatant was discarded. The remaining paste was re-suspended in a phosphate buffered DI water at pH of 7.0. This was to reduce the influence of the residual growth media on chlorine. Then the re-suspended algal biomass was transferred to clean and autoclaved glass bottles. Next, a solution of chlorine was added to the bottles to result in a desired dose. Then, the reactors were capped, and the contents were mixed continuously using stirrer plates. Samples were periodically collected from the reactors during the treatment process. Finally, the cell samples were characterized using cellometer and Scanning Electron Microscope (SEM) analyses, as appropriate.

The disruption experiment involving 10% algae paste was performed in clean VWR 50-mL centrifuge tubes. To the clean VWR tubes, a known mass of algae-paste and a solution of chlorine were added to achieve the desired dose and 10% algae cell concentration. The contents of the tubes were mixed using Heidolph UNimax 1010 shaker. Samples were withdrawn periodically and processed for lipid extraction using the Bligh and Dryer method [20], most widely used method for algal lipid extraction. During a typical lipid extraction process, 2 g of disrupted algae paste was transferred to 15-mL centrifuge tubes. Next, 4 mL of methanol and 2 mL of chloroform were added to the sample in the centrifuge tubes. Then the content of the tube was mixed for 2 min using a Thermolyne Maxi Mix Plus™ vortex (Dubuque, IA). Additional 2 mL of chloroform was added to the sample and the tube was mixed for 30 s using the vortex. Finally, 1.8 mL of DI water was added to the sample and then mixed for 30 s using the vortex. The lipid extraction process was performed at room temperature, in the range of 22 to 26 °C.

The mixture was centrifuged using Thermo Scientific Sorvall RC6+ centrifuge (Waltham, MA) at 10,000 G for 15 min. This provided complete separation with the chloroform/lipid layer at the bottom and the methanol/water layer on the top, while the residual biomass at the middle. The bottom chloroform layer was removed using a glass Pasteur pipette and placed into pre-weighed 125-mL Erlenmeyer flasks. The chloroform was evaporated from the flask using a Heidolph Hei-VAP Precision with glassware set to G5 rotary evaporator, with a bath temperature of 60 °C, pressure of 375 mbar, and rotation speed of 150 rpm.

### Materials

Chemicals and reagents used in the study were obtained from Fischer Scientific (Pittsburgh, PA) and Sigma-Aldrich Co. (St. Louis, Mo). For the project, *C. vulgaris*, one of the most widely researched algal species for biodiesel production, was used as representative microalgae. *C. vulgaris* culture was purchased from Carolina Biological Supply Company (Burlington, NC). Granular calcium hypocrite, purchased from Fisher Scientific, was used as source of chlorine.

#### *C. vulgaris* culture maintenance, cultivation and harvesting

*C. vulgaris* culture was grown in a medium prepared from MiracleGro All Purpose Water Soluble Plant Food. The media has been used in the past for the growth of microalgae [21].

During a typical growth cycle, few colonies of *C. vulgaris* from agar-plate cultures were aseptically transferred into 25 mL medium contained in 50 mL VWR tests tubes, and then capped with sponge plug. The test-tube cultures were placed under fluorescent lighting system, 14 h light and 10 h dark, and were aerated once a day using a vortex. After the culture growth reached to ~ 0.3 to 0.4 abs at 600 nm, it was transferred to eight 500 mL VWR Erlenmeyer flasks containing 350 mL medium. These cultures were aerated using aquarium air pump and were placed on stirrer plates for mixing and under the fluorescent light for 14 days.

Finally, after ~ 0.3 to 0.4 abs at 600 nm was achieved, the cultures were used for the inoculation of 3500 mL medium in eight 4 L VWR Erlenmeyer flasks. The cultures were aerated with an air stream containing 4.0% CO_2_ at a total flow rate of 200 mL/min or 25 mL/min per reactor. Reactors were placed on stirrer plates for mixing and under fluorescent light for 14 days. The cultures were harvested at an absorbance of ~ 0.5 at 600 nm. The cultures were concentrated with centrifugation at 10000 G for 10 min. A prior study in the PI’s lab has shown that this centrifugation force did not have any an impact on cell viability [22].

### Analytical methods

Algal cell concentrations (#cell/mL) and viabilities were determined optically via automated cell counts (Nexcellom Cellometer AutoX4). 20 μl of culture sample was combined with 20 μl propidium iodide (PI) stain (Cellometer ViaStain™ PI Staining Solution) in a 1.5 mL microcentrifuge tube and vortexed for 10 s. A 20 μl sample was then pipetted to a Cellometer counting chamber and allowed to stabilize for 2 min. A bright field cell was performed, followed by stimulation of the sample at 501 nm and emission measurement at 595 nm for 10 s of exposure. Dead cells were identified via fluorescence of PI, and an automated count of fluorescing cells were executed. Percent viability was then determined as the difference between the bright field and fluorescence cell counts divided by the bright field cell count. While cell diameter is directly measured by the Cellometer.

Absorbance at 600 nm was measured with Thermo Scientific BioMate™ 3S Spectrophotometer (Waltham, MA). The pH of the samples was measured using HACH-HQ440d Multi-Parameter Meter with HACH-IntelliCAL-pHC101 probe. Residual chlorine was measured according to Standard Methods [23].

SEM images of the algae cells were obtained using Quanta 450 FEG Scanning Electron Microscope (FEI, OR), housed in SDSU Electron Microscope Facility. For SEM analysis, algae samples treated with chlorine were fixed with a solution containing 4% (*v*/v) glutaraldehyde and 0.2 M Sodium Cacadolyic at pH 7.3. The cells were infiltrated by slowly washing with 0.1 M Sodium Cacadolyic at pH 7.3 and dehydrated using a graded ethanol concentration series of 30, 50, and 95% ethanol for 10 min each. Finally, the cells were soaked in 100% ethanol. A Critical Point Dryer was used to dry the samples further. The samples were then mounted on stubs and coated with platinum using a sputter coater. The morphologies of the surfaces of the ruptured cells were observed by SEM with an accelerating voltage of 5.0 kV.

## Results and discussion

### Disruption of algae cells in suspension

Several experiments were conducted to investigate the disruption of algae cells in suspension using chlorine as an agent. On average, the concentration of algae in the feedstock was 0.02% (0.2 g/L) on dry mass basis. The chlorine doses were 0 (control), 5, 6.5, 8, and 10 mg/L as Cl_2_. Samples were periodically collected from the reactors during treatment process at 0, 3, 6, 15, 30, 45, and 60 min.

**Fig. 1:**
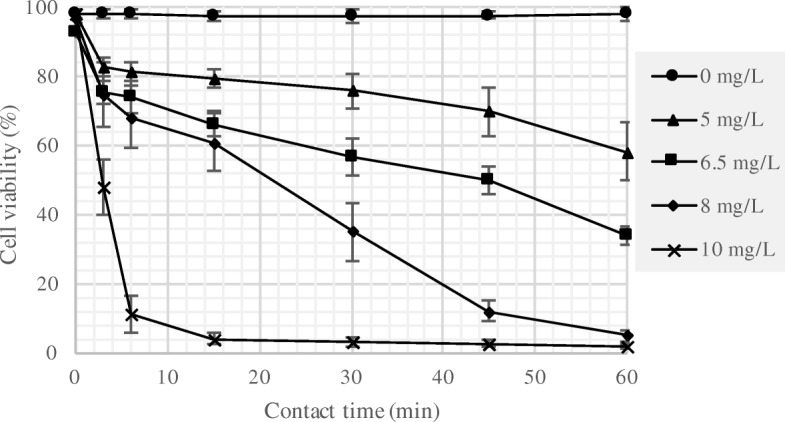
Algal cell viability at varying chlorine dose and contact times, and the results are presented as the means ± SD

**Fig. 2:**
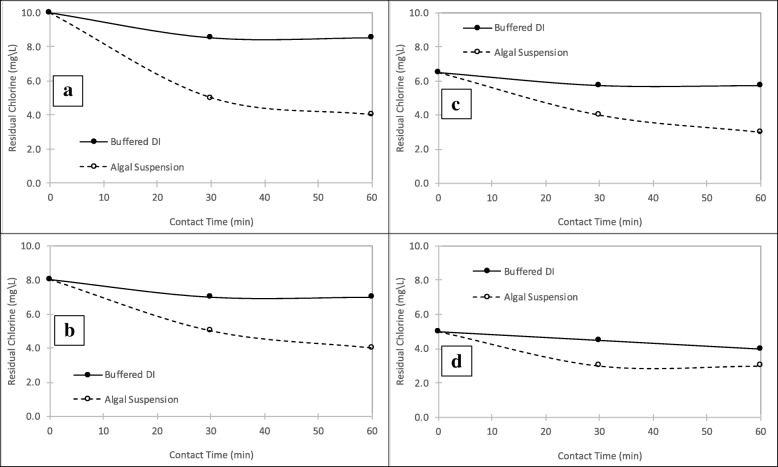
Chlorine residual for algal suspension and buffered DI water at: (**a**) 10 mg/L chlorine dose, (**b**) 8 mg/L chlorine dose, (**c**) 6.5 mg/L chlorine dose, and (**d**) 5 mg/L chlorine dose

The cell viability results from the cellometer analysis are presented in Fig. 1. The data represents mean values from sextuplicate analysis, from triplicate reactors, with one standard deviations (SD) above and below the mean. The results revealed that the cell viability for the reactors dosed with 0 mg/L of chlorine (controls) remained constant at ~ 100% over the course of the treatment process as expected, while the cell viabilities decreased for samples dosed with 5, 6.5, 8 and 10 mg/L chlorine as Cl_2_. For the samples dosed at 10 mg/L of chlorine, the cell viability decreased to about 10% in 6 min, while for the other dose levels the decrease in cell viability was gradual. The decrease in cell viability was directly proportional to the quantity of chlorine consumed. The chlorine residual for the algal suspensions and control samples is presented in Fig. 2. In controls, a phosphate buffered DI water was used to estimate the chlorine demand for the suspension. The results showed that about 1.0, 2.75, 3.0, and 4.5 mg/L of chlorine was consumed at the end of one-hour contact time by algal suspensions dosed with 5, 6.5, 8, and 10 mg/L of chlorines, respectively.

**Fig. 3:**
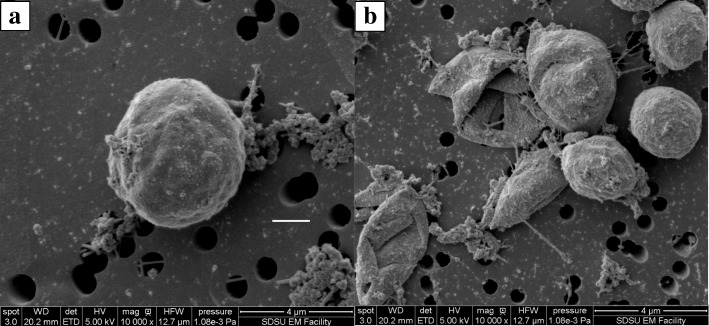
Scanning electron micrographs of the *C. vulgaris* cell surfaces: (**a**) cell without treatment (control) and (**b**) cells treated with chlorine

After treatment with chlorine, the surfaces of the ruptured cells were analyzed with SEM. The morphologies of the surfaces are shown in Fig. 3. Figure 3a shows the morphology of the *C. vulgaris* cell surface without treatment, from control reactors. Figure 3b shows the morphologies of *C. vulgaris* cells treated with chlorine, illustrating the broken appearance of the cells into irregular shapes, compared to the spherical for non-treated (control) cells in Fig. 3a. In addition, cells treated with chlorine exhibited uneven shrinkages and creases.

The mechanism of action, how chemical agents act to inhibit or kill cells, can differ widely, and may include: disruption of cell wall and cell membrane, inhibition of respiration or metabolic reactions, disruption of cell division, and DNA damage, among others [24]. There are no known or proposed mechanisms by which chlorine reacts with algae cells in the literature. Moreover, the mechanism by which chlorine kill or inhibit the growth of microorganisms is not clearly understood, even though it has been used as a disinfectant for several decades. The majority of chlorine disinfection research, conducted prior to 1980s, focused on bacteria, and speculated that chlorine reacts with biomolecules in the bacteria cell, such as proteins [25, 26], sulfhydryl [27], and α-amino acids [28], leading to oxidation, hydrolysis, and/or deamination reactions that destroy the bacteria. More recent studies postulated that bacterial death probably results from chlorine attacking a variety of biomolecules or targets [29–32], including enzymatic activities, nucleic acids, DNA, and membrane lipids.

**Fig. 4:**
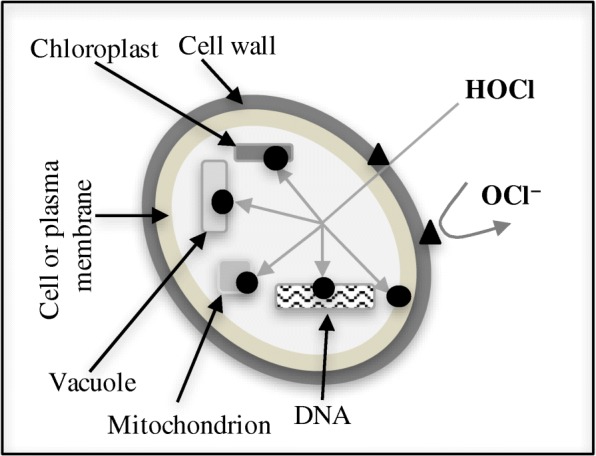
A proposed model for mechanism of actions of HOCl and OCl − against algae cell

For the reaction of chlorine with algae cell, we proposed a model (Fig. 4) similar to the mechanism of actions of chlorine against bacteria proposed by Fukuzaki [29]. In the model, a “multiple attack” theory for the disruption of algae cell is proposed. As disinfectant, chlorine is applied as chlorine gas or in the form of hypochlorite solutions. When chlorine gas is used, it reacts with water to form HOCl, which in turn dissociates into OCl^−^ and hydrogen ion (H^+^). When NaOCl or Ca(OCl)_2_ are used as disinfectants, they will dissolve in water resulting in OCl^−^, which in turn reacts with water to form HOCl and OH^−^. The HOCl and OCl^−^ species, commonly referred to as free chlorine, are strong oxidizing agents and could react with the algae cell wall as shown in Fig. 4 (triangles). Algae cell walls are comprised of either polysaccharides, or glycoproteins, or both [33]. Polysaccharides are polymeric carbohydrate molecules composed of long chains of monosaccharide units bound together by glycosidic linkages, while glycoproteins are proteins that contain a few monosaccharide chains covalently attached to polypeptide side-chains. Previous studies have shown that polysaccharides and glycoproteins contained in aqueous solutions are effectively depolymerized using chlorine [34, 35], supporting the premise of this project.

Algae cell or plasma membrane is composed of a bilayer of phospholipid molecules [36], which carry a net negative charge [37], serving as a barrier to OCl^−^ entry into the inside of the algae cell. This will limit the mechanism of actions of OCl^−^ to the algae cell wall. On the other hand, HOCl can penetrate the lipid bilayer, due to its electrical neutrality and its comparable molecular size to that of water. The molecular radius of water is ~ 0.76 Å [38], while that of HOCl is ~ 1.06 Å [39]. Therefore, HOCl can also react with cell constituents as shown in the Fig. 4 (circles).

**Fig. 5:**
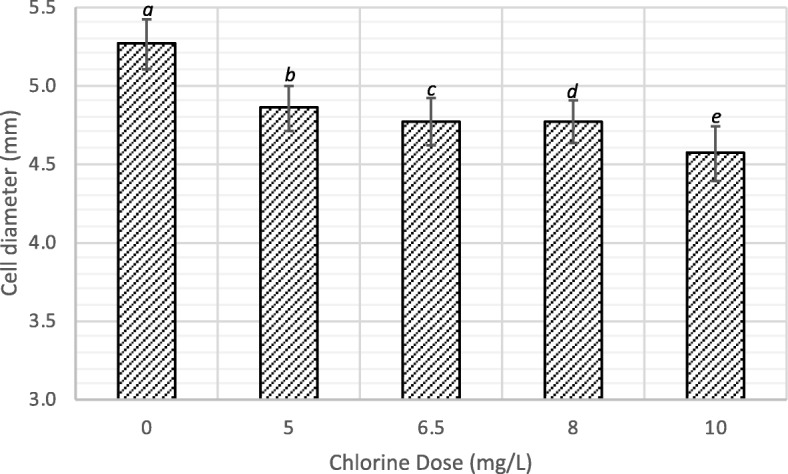
Algal cell diameter with varying chlorine dose at the end of 60-min contact time, and the results are presented as the means ± SD. Different italicized letters (*a-e*) indicate significant differences (*P* < 0.05)

The cell disruption studies were conducted at around neutral pH, where the concentrations of HOCl and OCl^−^ would be significant, and therefore, both species of chlorine are expected to contribute to the cell disruption process. From the SEM morphologies of the disrupted cells, it can be deduced that the cell breakages observed may have been caused by the reaction of HOCl and OCl^−^ with the algae cell wall. The cell shrinkages observed may have been resulted from the penetration of the algal cell wall by HOCl and subsequent reaction with the cell constituents. The dimeter of the algal cells decreased with increase in the concentration of chlorine, Fig. 5. This confirms the cell shrinkages observed with SEM analysis.

### Disruption of algae cells in paste

**Fig. 6:**
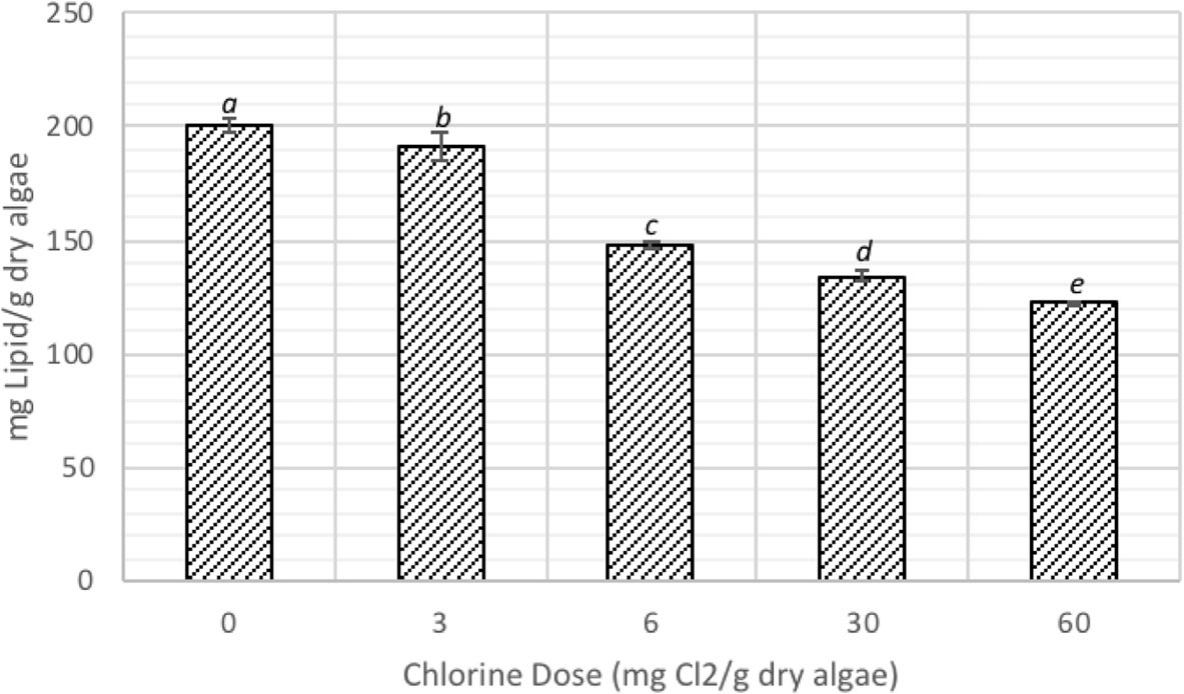
Lipid extraction yield with varying chlorine dose, and the results are presented as the means ± SD. Different italicized letters (*a-e*) indicate significant differences (*P* < 0.05)

As shown in the results presented in the previous subsection, disruption experiment conducted with a dose of 10 mg/L chlorine resulted in the highest percentage of dead cells. This dose results in 50 mg of chlorine per g of dry algae; the algal biomass concentration in the feedstock was 0.2 g/L on dry mass basis. Accordingly, disruption of algal biomass was conducted using 10% algae paste dosed with 0 (controls), 3, 6, 30, and 60 mg of chlorine as Cl_2_ per g of dry algae. At the end of 30-min of contact time, lipid extraction was performed on the samples. The results as mg of lipid extracted per g of dry algae are presented in Fig. 6.

The data revealed that lipid extraction decreased with chlorine dose, indicating that chorine reacted with and mineralized the cell contents. For control samples, the lipid extraction yield remained constant around 200 mg of lipid per g of *C. vulgaris*. The lipid extraction decreased to 192, 148, 135, and 122 mg of lipid per g of *C. vulgaris* for samples dosed with 3, 6, 30, and 60 mg of chlorine per g of dray algae, respectively. These are equivalent to 4, 26, 33, and 40% decrease in lipid extraction yield compared to the control for samples dosed at 3, 6, 30, and 60 mg of chlorine per g of dry algae, respectively.

It is also anticipated that the residual chlorine may affect the integrity of other components, proteins and carbohydrates, remaining in the residual biomass once the lipids are extracted.

### Energy-efficiency, cost-effectiveness, and sustainability of chlorine

As shown in the previous sections, chorine was effective in disrupting algae cells. However, it was observed that the residual chlorine reacted with and mineralized the cell contents, which is undesirable. Is this the end of the road for chlorine as a potential cell-disruption agent? Is it necessary to undertake a research effort to address this challenge? The authors believe the latter is reasonable; however, before taking such research efforts, preliminary analyses on the energy-efficiency, cost-effectiveness, and sustainability of chlorine as algal biomass disruption agent should be performed using the data collected in this study and readily available in the literature. In the following paragraphs, an attempt was made to answer these questions.

In the U.S. and Europe, chlorine is primarily produced from the electrolysis of a brine solution, and the process also results roughly in equivalent amounts of sodium hydroxide, i.e., about 1.1 tons of sodium hydroxide for every ton of chlorine produced. Hydrogen is also produced in equal molar amounts with chlorine and sodium hydroxide. Currently, diaphragm cells, mercury cells, and membrane cells are used to keep the chlorine produced at the anode separated from the sodium hydroxide and hydrogen produced at the cathode. The net energy consumption by three methods was estimated as 10.12, 12.15, and 10.07 MJ/kg of chlorine produced [40], with an average value of 10.78 MJ/kg of chlorine.

For cell disruption experiments conducted using algal biomass in suspension, a dose of 10 mg/L chlorine resulted in the highest percentage of dead cells (Fig. 1). This dose resulted in 50 mg of chlorine per g of dry algae; the algal biomass concentration in the feedstock was 0.2 g/L on dry mass basis. Therefore, the embedded specific energy required for disrupting algae cells with chlorine was estimated as 0.54 MJ/kg dry algae.

It could be argued that the total energy requirements for chlorine production and transportation from the production site to the end use location must be considered, particularly in comparison with mechanical methods that have almost no supply chain energy costs after installations. A study that evaluated the environmental impacts of chlorine for the water and wastewater industry reported that the total energy for chlorine as 74.55 MJ/kg chlorine [41]. Assuming that algal biofuel facilities are located at about the same distance from chlorine production sites as water and wastewater facilities, the total specific energy required for disrupting algal biomass with chlorine was estimated as 3.73 MJ/kg dry algae. In Table 1, the specific energy requirement for chlorine estimated in this study and for other algal biomass disruption methods reported in the literature [8] are presented. The specific energy for chlorine was significantly less than the requirement for hydrodynamic cavitation, 33 MJ per kg of dry algae, which is the most “efficient” of the existing algae cells disruption methods. The specific energy requirements for the other methods are significantly higher than that of chlorine; for instance, pulsed electric field has over 230 times the requirement for chlorine.

**Table 1:** Specific energy requirement and global warming potential of selected algal biomass disruption methods

Algal cell disruption methods	Energy use MJ/kg dry mass	GWP (kg CO_2_-eq./kg dry algae)
Chlorine (this study)	3.73	0.3
Hydrodynamic cavitation	33	5.9
High speed homogenizer	67.5	12.2
Sonication	132	23.8
Freeze drying	140	25.2
Microwave	420	75.6
Bead mills	504	90.7
High pressure homogenizer	529	95.2
Pulsed electric field	860	154.8

The specific energy for the existing algal cell disruption methods were obtained from literature [8], and the rest of the data in the table was estimated in this study

The Global Warming Potential (GWP) for chlorine used at water and wastewater facilities was estimated as 6.13 kg CO_2_-eq./kg of chlorine [41], including contributions from chlorine production and transportation. Based on chlorine dose 50 mg/g dry algae, the GWP of chlorine used for algal biomass disruption was estimated as 0.3 kg CO_2_-eq./kg dry algae. On the other hand, the average GWP of electricity generation from coal and natural gas, which account for 66% U.S. electricity generation [42], was estimated as 180 g CO_2_-eq./MJ of electricity generated [43]. For hydrodynamic cavitation, the GWP was estimated as 5.9 kg CO_2_-eq./kg dry algae (Table 1); significantly higher than that of chlorine. Similarly, the estimated GWPs for the other disruption methods were significantly higher than that of chlorine, Table 1.

Based on data obtained from a local water treatment plant based in San Diego, California, the price for liquid chlorine was estimated at 60 cents per gallon or 10.8 cents per kg; density of chlorine is 1.467 kg/L. Using the chlorine dose estimated above, one gallon of liquid chlorine can be used for disrupting ~ 110 kg of dry algae. The additional cost due to the use of chlorine was estimated at ~ 0.54 cents per kg of dry algal cells disrupted. This is significantly less than the savings from reductions in energy use from 33 MJ (for hydrodynamic cavitation) to 3.73 MJ (total energy for chlorine) per kg of dry algae. Using a retail energy price of 10 cents per kilowatt-hour, the reductions in energy use of 29.27 MJ corresponds to ~ 80 cents saving per kg of dry algal cells disrupted.

### Recommended future work

This study showed that chlorine was effective in disrupting algae cells. The estimated specific energy requirement, specific cost, and GWP clearly demonstrated that chlorine has a potential to be developed into an energy-efficient, cost-effective and sustainable method for algae biomass disruption. However, it was observed that the residual chlorine reacted with and mineralized the cell contents, which is undesirable. Therefore, future research efforts must be focused on addressing this challenge. One of such research efforts could be developing ways to minimize the contact between the residual chlorine and the cell contents after algal biomass disruption. This may be achieved by immobilizing chlorine on surface and then allowing the algal suspension to come in contact with the surface in a flow-through system.

The residual chlorine may also be quenched to minimize its reaction with the cell contents. Some of the most commonly used chemical quenchers in the water and wastewater industry, sodium bisulfate, ascorbic acid, sodium sulfite, and sodium thiosulfate, could be investigated for this purpose.

## Conclusions

Chlorine was effective in disrupting algae cells. Moreover, the estimated specific energy requirement, specific cost, and GWP for chlorine were less than those for the existing algal biomass disruption methods. The estimated specific energy requirement for chlorine was 3.73 MJ/kg of dry algae disrupted compared to 33 MJ/ kg of dry algae for hydrodynamic cavitation, the most energy-efficient of the existing methods. The GWP for chlorine was estimated as 0.3 kg CO_2_-eq./kg dry algae disrupted, while for the existing methods it varied from 5.9 to 369.8 CO_2_-eq./kg dry algae.

Despite these advantages, it was observed that residual chlorine mineralized the cell contents. Therefore, future research efforts must be focused on addressing this challenge. One of such research effort is exploring the possibility of immobilizing chlorine on a surface, where algae cells come in contact with the surface in a flow-through system. Moreover, the quenching of the residual chlorine could be explored as a future research effort. If the reaction of chlorine with the cell contents is minimized or eliminated as part of the future research efforts, then chlorine has a potential to be developed into an energy-efficient, cost-effective, and sustainable method for algae cells disruption.

## Abbreviations

DI: Deionized
DNA: Deoxyribonucleic Acid
EROI: Energy Return of Investment
GWP: Global Warming Potential
PI: Propidium Iodide
PTFE: Polytetrafluoroethylene
SD: Standard Deviation
SEM: Scanning Electron Microscope
